# Impact of CTO-related myocardial infarction and procedural success on long-term outcomes after CTO PCI

**DOI:** 10.3389/fcvm.2026.1846597

**Published:** 2026-07-28

**Authors:** HunJoo Lee, YouMi Hwang, Sung Jung Kim, Myungjae Yoo

**Affiliations:** 1 Department of Cardiology, St. Vincent's Hospital, The Catholic University of Korea, Suwon, Republic of Korea; 2 Catholic Research Institute for Intractable Cardiovascular Disease (CRID), College of Medicine, The Catholic University of Korea, Seoul, Republic of Korea

**Keywords:** chronic kidney disease, chronic total occlusion, long-term outcomes, myocardial infarction, percutaneous coronary intervention, procedural success

## Abstract

**Background:**

The prognostic impact of CTO-related myocardial infarction (MI) and procedural success on long-term outcomes after chronic total occlusion (CTO) PCI remains uncertain. This study evaluated clinical and procedural predictors of adverse outcomes following CTO PCI.

**Methods:**

In this retrospective registry, 347 patients who underwent CTO PCI were analyzed after screening 543 individuals between 2004 and 2018—CTO-related MI and procedural success stratified patients. The primary composite endpoint included ventricular tachyarrhythmia and follow-up MACCE, defined as cardiopulmonary resuscitation, coronary artery bypass grafting, pacemaker or implantable cardioverter-defibrillator implantation, acute coronary syndrome, cardiovascular death, or all-cause death. Kaplan–Meier analysis and bootstrap-derived Cox models were used to assess long-term risk.

**Results:**

CTO-related MI was associated with a higher cumulative incidence of adverse outcomes, whereas procedural success was associated with improved event-free survival. In multivariable analysis, chronic kidney disease was the strongest independent predictor of the composite endpoint, while higher left ventricular ejection fraction was protective. Procedural success showed a trend toward lower risk, whereas CTO-related MI showed a trend toward higher risk in multivariable analysis.

**Conclusions:**

CTO-related MI and unsuccessful CTO PCI were associated with worse long-term outcomes. Chronic kidney disease was the dominant independent predictor, whereas preserved ventricular function was protective. These findings highlight the prognostic value of integrating clinical risk factors and procedural success to refine long-term management strategies after CTO PCI.

## Introduction

Chronic total occlusion (CTO) is a commonly observed lesion in patients with coronary artery disease, characterized by long-standing arterial blockage that can lead to ischemia and structural remodeling within the affected myocardial territory. These pathophysiological changes have been associated with impaired left ventricular function, an increased risk of ventricular arrhythmias, and potentially higher long-term mortality (1, 2). However, patients with CTO exhibit considerable clinical and anatomical heterogeneity, which complicates the establishment of consistent treatment strategies and prognostic predictions (3).

Percutaneous coronary intervention (PCI) has been employed in CTO patients to improve myocardial perfusion and alleviate symptoms, and advances in interventional techniques and devices have progressively enhanced procedural success rates. Nonetheless, long-term clinical outcomes following CTO PCI are influenced by multiple factors, and the pattern of adverse events during follow-up may vary depending on procedural results (4). Consequently, interpretation of long-term prognosis after CTO PCI requires consideration of both procedural success and the patient's baseline clinical status.

A subset of CTO patients has a history of myocardial infarction (MI) in the territory of the occluded vessel, suggesting the presence of irreversible myocardial damage and electrical substrate alterations (5). The impact of CTO revascularization on long-term outcomes in these patients, particularly regarding ventricular arrhythmias and major cardiovascular events, has not been fully elucidated (6).

Chronic kidney disease (CKD) is a well-recognized adverse prognostic factor in patients with coronary artery disease. In the context of CTO PCI, data on the relative influence of CKD on long-term outcomes, when considered alongside procedural success and ventricular function, remain limited. CKD may contribute to both ischemic events and arrhythmogenesis through systemic vascular disease, metabolic abnormalities, and heightened inflammatory responses, highlighting its potential importance in risk stratification of CTO patients (7).

Against this background, the present study aimed to evaluate the relationship between clinical factors—including prior MI in the CTO territory, CKD, and left ventricular function—procedural outcomes, and long-term clinical prognosis in patients undergoing CTO PCI. Using a composite endpoint encompassing ventricular arrhythmias and major cardiovascular and cerebrovascular events during follow-up, this study seeks to provide a comprehensive assessment of long-term outcomes after CTO PCI.

## Methods

### Study design and population

**Figure 1:**
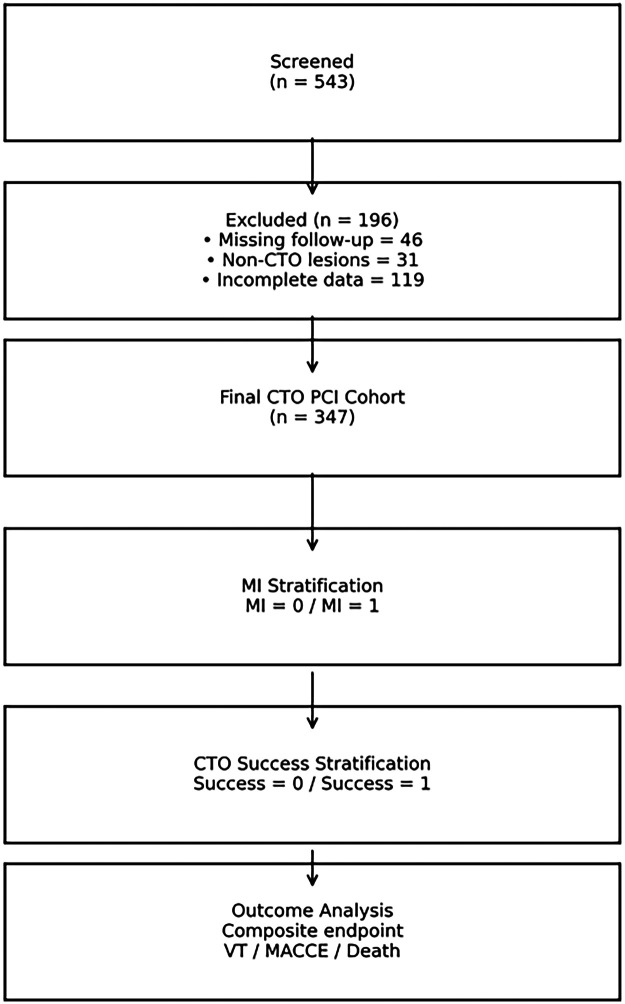
Study flow diagram. Study flow diagram of the retrospective single-center CTO registry. Of 543 patients screened for suspected chronic total occlusion, 347 who underwent CTO percutaneous coronary intervention (PCI) and had available clinical and follow-up data were included in the final analytic cohort.

This study was conducted as a retrospective, single-center registry including consecutive patients who underwent evaluation for chronic total occlusion (CTO) at our institution. A total of 543 patients who received coronary angiography with suspected CTO between 2004 and 2018 were screened for eligibility. Patients were excluded if they had: (1) missing longitudinal follow-up information, (2) angiographically confirmed non-CTO lesions, or (3) incomplete clinical or procedural data. After applying these criteria, 347 patients who underwent CTO percutaneous coronary intervention (PCI) were included in the final analytic cohort (Figure 1). Patients were stratified by the presence of CTO-related myocardial infarction (MI) and by procedural success of CTO PCI. The study protocol adhered to the Declaration of Helsinki and was approved by the institutional review board (VC20RISI0242), with informed consent waived due to the retrospective nature of the analysis.

### Data collection

Clinical, laboratory, procedural, and follow-up data were obtained from the institutional electronic medical record system and catheterization laboratory database. Demographic characteristics, cardiovascular risk factors (hypertension, diabetes mellitus, smoking status, chronic kidney disease, peripheral artery disease, chronic obstructive pulmonary disease, and prior cerebrovascular accident), and cardiac biomarkers were collected at the index hospitalization. Angiographic characteristics, including CTO location, collateral grade, and procedural parameters, were reviewed by two experienced interventional cardiologists. Follow-up information—including arrhythmic events, device implantation (PPM or ICD), coronary revascularization (PCI or CABG), acute coronary syndrome (ACS), cardiovascular death, and all-cause death—was obtained from clinic visits, hospital records, and national mortality databases. Follow-up duration was calculated as the interval from the index CTO PCI date to the last clinical encounter or death. A comprehensive review of electronic medical records and physician documentation adjudicated all outcomes.

### Definitions of variables

Hypertension was defined as a prior diagnosis or current use of antihypertensive medication. Diabetes mellitus was defined as a known diagnosis, hemoglobin A1c ≥ 6.5%, or treatment with oral hypoglycemic agents or insulin. Chronic kidney disease (CKD) was defined as an estimated glomerular filtration rate <60 mL/min/1.73 m^2^ (CKD stage ≥3) or a documented diagnosis of CKD. Left ventricular ejection fraction (LVEF) was obtained from transthoracic echocardiography performed within 6 months of the index PCI. CTO-related MI was defined as a prior MI attributable to the vascular territory supplied by the target CTO vessel, based on clinical presentation, biomarker elevation, and corresponding ECG or imaging abnormalities. Procedural success of CTO PCI was defined as final residual stenosis <30% with restoration of TIMI grade 3 antegrade flow.

### Definitions of outcomes

The primary composite endpoint was selected to capture the heterogeneous but clinically significant adverse event spectrum in this CTO cohort, including arrhythmic, ischemic, and fatal events. Given the modest sample size and long follow-up, a composite endpoint was chosen to preserve statistical power. To address the heterogeneity of the composite, prespecified secondary analyses examined all-cause mortality and cardiovascular death separately. Follow-up MACCE was defined as cardiopulmonary resuscitation (CPR), coronary artery bypass grafting (CABG), pacemaker implantation (PPM), implantable cardioverter-defibrillator implantation (ICD), acute coronary syndrome (ACS), cardiovascular death, or all-cause death. Ventricular tachyarrhythmia included documented sustained ventricular tachycardia or ventricular fibrillation recorded on telemetry, Holter monitoring, ICD interrogation, or in-hospital rhythm documentation. Secondary endpoints included the individual components of the composite endpoint and exploratory analyses of ventricular arrhythmia occurrence.

### Statistical analysis

All statistical analyses were performed using standard methodology for observational studies. Continuous variables were summarized as medians with interquartile ranges (IQRs), and categorical variables as counts and percentages. Between-group comparisons were conducted using the Mann–Whitney *U*-test for continuous variables and the *χ*^2^ or Fisher's exact test for categorical variables, as appropriate.

Time-to-event outcomes were analyzed using Kaplan–Meier survival curves, and differences between groups were assessed with the log-rank test. Follow-up duration was calculated as the interval from the index CTO PCI date to the occurrence of the composite endpoint or to the most recent clinical encounter, whichever occurred first. The composite endpoint consisted of ventricular tachyarrhythmia and follow-up MACCE, defined as cardiopulmonary resuscitation (CPR), coronary artery bypass grafting (CABG), pacemaker implantation (PPM), implantable cardioverter-defibrillator implantation (ICD), acute coronary syndrome (ACS), cardiovascular death, or all-cause death.

To identify independent predictors of the composite endpoint, Cox proportional hazards models were constructed. To ensure clinical relevance and avoid model overfitting, four prespecified covariates were included in the multivariable model: prior myocardial infarction, successful CTO PCI, left ventricular ejection fraction (LVEF), and chronic kidney disease (CKD).

Because the event distribution indicated sparsity and potential separation—particularly among patients with CKD—conventional maximum-likelihood estimation yielded unstable variance estimates. Therefore, nonparametric bootstrap resampling (1,000 iterations) was used to obtain robust percentile-based 95% confidence intervals for the regression coefficients. Hazard ratios (HRs) were derived from the fitted initial model.

All analyses were performed using R statistical software (version 4.5.2; R Foundation for Statistical Computing, Vienna, Austria). Proportional hazards assumptions were evaluated using log–log survival plots and were not violated. A two-sided *p*-value < 0.05 was considered statistically significant.

## Results

### Study population and flow

A total of 543 patients were screened in this retrospective registry. Of the 543 patients initially screened, 196 were excluded for the following reasons: 46 had inadequate longitudinal follow-up (defined as no documented clinical encounter or mortality record beyond 30 days from the index procedure); 31 were found on chart review to have angiographically confirmed non-CTO lesions; and 119 had incomplete clinical or procedural data, most often missing echocardiographic data (principally LVEF) or missing laboratory values relevant to CKD classification. (Figure 1). Patients were stratified by history of CTO-related myocardial infarction (MI) and procedural success (Supplementary Table S1).

### Baseline characteristics

Baseline clinical characteristics according to MI status are summarized in Table 1. Patients with CTO-related MI had substantially lower left ventricular ejection fraction (LVEF) than those without MI. In contrast, most other demographic and clinical variables, including age, sex, hypertension, diabetes mellitus, smoking status, and chronic kidney disease, were generally comparable between groups.

**Table 1:** Baseline characteristics.

Variable	MI = 0 (*n* = 241)	MI = 1 (*n* = 106)
Age (years)	62.00 [55.00–72.00]	64.00 [55.00–75.00]
Sex—Male, *n* (%)	170 (70.5%)	70 (66.0%)
Sex—Female, *n* (%)	71 (29.5%)	36 (34.0%)
Hb (g/dL)	13.60 [12.07–14.80]	13.90 [12.35–15.07]
Platelet (×10^3^/µL)	225.50 [191.75–259.00]	224.00 [185.00–261.50]
BUN (mg/dL)	16.05 [13.40–20.55]	17.75 [13.80–22.68]
Creatinine (mg/dL)	0.90 [0.80–1.20]	1.00 [0.90–1.20]
LDL-C (mg/dL)	106.00 [82.75–136.50]	106.50 [77.50–133.25]
Triglyceride (mg/dL)	111.00 [77.00–178.50]	94.00 [63.00–139.00]
CRP (mg/L)	0.20 [0.10–0.75]	0.50 [0.10–4.38]
LVEF (%)	58.30 [48.00–63.10]	47.80 [37.10–58.20]
Hypertension, *n* (%)	154 (63.9%)	63 (59.4%)
Diabetes Mellitus, *n* (%)	98 (40.7%)	41 (38.7%)
Smoking, *n* (%)	101 (41.9%)	48 (45.3%)
CKD, *n* (%)	23 (9.5%)	7 (6.6%)
PAD, *n* (%)	2 (0.8%)	0 (0.0%)
COPD, *n* (%)	4 (1.7%)	0 (0.0%)
CVA history, *n* (%)	20 (8.3%)	12 (11.3%)

Baseline clinical characteristics of the study population according to the presence of CTO-related myocardial infarction (MI). Continuous variables are presented as median [interquartile range], and categorical variables as counts and percentages. MI, myocardial infarction; Hb, hemoglobin; BUN, blood urea nitrogen; LDL-C, low-density lipoprotein cholesterol; CRP, C-reactive protein; LVEF, left ventricular ejection fraction; CKD, chronic kidney disease; PAD, peripheral artery disease; COPD, chronic obstructive pulmonary disease; CVA, cerebrovascular accident.

### Incidence of the composite outcome

**Figure 2:**
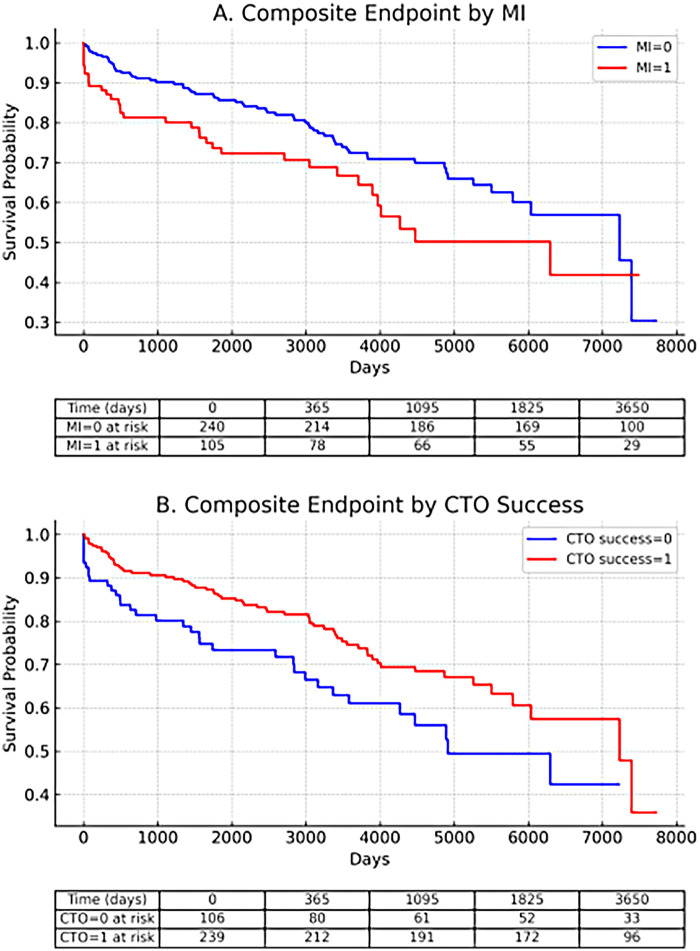
Kaplan–meier analysis of long-term outcomes. Kaplan–Meier curves for event-free survival according to **(A)** the presence of CTO-related myocardial infarction and **(B)** procedural success of CTO PCI. Event-free survival was defined as freedom from ventricular tachyarrhythmia and follow-up major adverse cardiovascular and cerebrovascular events (MACCE). Differences between groups were assessed using the log-rank test.

The median follow-up duration was 9.4 years (interquartile range, 4.8–13.1 years). During the follow-up period, the composite outcome—defined as ventricular tachyarrhythmia and follow-up MACCE (CPR, CABG, PPM implantation, ICD implantation, ACS, cardiovascular death, or all-cause death)—was assessed. Patients with CTO-related MI had a higher cumulative incidence of the composite outcome than those without MI. In contrast, patients with successful CTO PCI had a markedly lower incidence than those with CTO PCI failure (Figure 2).

### Kaplan–Meier survival analysis

Kaplan–Meier curves showed significantly lower event-free survival in patients with CTO-related MI than in those without MI (Figure 2A). Successful CTO PCI was associated with improved long-term survival and fewer composite events than unsuccessful procedures (Figure 2B). Numbers at risk at prespecified time points are presented beneath each panel. Procedural success was associated with lower all-cause mortality (*p* = 0.025) and cardiovascular death (*p* = 0.015), while the association with CTO-related MI status did not reach significance for either hard endpoint. This pattern is consistent with our primary composite endpoint analysis and, importantly, suggests that procedural success is associated with improved hard clinical outcomes in unadjusted analyses.

### Multivariable Cox regression

A multivariable Cox proportional hazards model including CTO-related MI, CTO PCI success, LVEF, and CKD identified CKD as the strongest independent predictor of the composite outcome (HR 3.63, 95% CI 2.00–6.09). Higher LVEF was associated with a reduced risk (HR = 0.97; 95% CI, 0.96–0.99). Successful CTO PCI showed a trend toward lower event risk that did not reach statistical significance (HR 0.67, 95% CI 0.46–1.04), while CTO-related MI demonstrated a nonsignificant trend toward increased risk (HR 1.35, 95% CI 0.87–2.16). Multivariable predictors of the composite endpoint are summarized in Table 2 and Supplementary Figures S1 and S2. Given the small number of events observed in CKD, we performed a Kaplan–Meier analysis of the composite endpoint stratified by CKD status, comparing successful and unsuccessful CTO PCI within each stratum, and then tested a CKD × procedural success interaction term in the Cox model as a sensitivity analysis (Supplementary Figure S3).

**Table 2:** Multivariable Cox regression analysis.

**Variable**	**Hazard ratio (95% CI)**
MI	1.35 (0.87–2.16)
Successful CTO PCI	0.67 (0.46–1.04)
LVEF, per 1% increase	0.97 (0.96–0.99)
Chronic kidney disease	3.63 (2.00–6.09)

Multivariable Cox proportional hazards analysis for the composite endpoint. Hazard ratios (HRs) and 95% confidence intervals (CIs) were derived using nonparametric bootstrap resampling (1,000 iterations). Variables were selected *a priori* based on clinical relevance. CTO, chronic total occlusion; PCI, percutaneous coronary intervention; LVEF, left ventricular ejection fraction; CKD, chronic kidney disease.

### Summary of findings

Overall, successful CTO PCI was associated with improved long-term outcomes and a lower incidence of major clinical events. Chronic kidney disease emerged as a dominant independent risk factor, whereas CTO-related MI and LVEF contributed meaningfully to risk stratification. These findings underscore the prognostic importance of both anatomical (CTO success) and clinical (CTO-related MI, CKD, LVEF) characteristics in patients with CTO.

## Discussion

In this retrospective registry of patients undergoing PCI for chronic total occlusion, several key observations were identified. First, the presence of CTO-related myocardial infarction was associated with a markedly higher incidence of long-term adverse outcomes, including ventricular tachyarrhythmia and follow-up MACCE (5, 8). Second, procedural success in CTO PCI was associated with a trend toward lower clinical event risk, as reflected by improved event-free survival on Kaplan–Meier analysis (9). Third, in multivariable modeling, chronic kidney disease emerged as the strongest independent predictor of the composite endpoint, whereas higher left ventricular ejection fraction conferred a protective effect (10). Together, these findings highlight the importance of both clinical phenotype and procedural factors in determining long-term risk after CTO PCI (11).

### Interpretation of CTO-related MI and procedural success

CTO-related MI may reflect a distinct clinical subset characterized by impaired myocardial viability and a higher susceptibility to arrhythmogenic events (5, 8). The higher event rates observed in this group likely reflect a combination of structural myocardial damage and vulnerability to subsequent ischemic or electrical complications. Successful recanalization, by contrast, was associated with a lower cumulative incidence of adverse events, underscoring the prognostic value of restoring antegrade flow in the CTO territory (9, 12). Patients in the unsuccessful CTO PCI group had significantly lower baseline LVEF and a numerically higher serum creatinine, although the prevalence of CKD and most other comorbidities did not differ significantly between groups. The association between procedural failure and adverse outcomes may therefore reflect, in part, residual baseline differences in left ventricular function rather than an independent prognostic effect of unsuccessful revascularization. This caveat is consistent with the borderline hazard ratio for procedural success observed in our multivariable model. Importantly, LVEF was included in the multivariable model, which attenuated the association between procedural success and outcome. Although the association was not statistically significant in the bootstrap-adjusted Cox analysis, the consistent trend across survival curves suggests that procedural success may mitigate long-term risk in selected patients (11, 13).

### Role of CKD and LVEF in risk stratification

Among the evaluated covariates, chronic kidney disease demonstrated the strongest association with adverse outcomes, with more than a threefold increase in risk (14). This finding emphasizes the substantial impact of impaired renal function on cardiovascular prognosis in the CTO population (15). Event-free survival according to CKD status is shown in Supplementary Figure S3. CKD may amplify susceptibility to both arrhythmic and ischemic complications, contributing to the elevated burden of follow-up MACCE observed in this cohort (10). In contrast, preserved left ventricular ejection fraction was associated with improved outcomes, supporting its role as an important determinant of long-term clinical stability (9). A prespecified interaction between CKD and procedural success was not statistically significant (HR 2.94, 95% CI 0.72–11.99, *p* = 0.265). However, the very small number of patients with CKD (*n* = 28) limits the precision of this estimate, and the apparent lack of benefit from successful CTO PCI in CKD patients should not be interpreted as evidence of effect modification in the absence of larger confirmatory studies. Together, CKD and LVEF provide valuable complementary information for risk stratification beyond anatomical considerations.

### Clinical implications

The present findings hold practical relevance for clinicians managing patients with CTO. Identification of CTO-related MI, reduced LVEF, or underlying CKD at baseline may help flag individuals at heightened risk for subsequent ventricular arrhythmia or major cardiac events (5, 9, 14). Furthermore, efforts to optimize procedural strategies and enhance the likelihood of successful recanalization may offer clinical benefit in appropriately selected patients (9, 12). Incorporating these clinical and procedural markers into routine post-PCI assessment could facilitate more tailored follow-up strategies and more informed discussions regarding prognosis.

### Limitations and concluding remarks

This study has limitations inherent to its retrospective, single-center design, including potential selection bias and unmeasured confounding. Event adjudication was based on electronic medical records, which may not capture events occurring outside the institution. A substantial proportion of patients screened (119 of 543; 22%) were excluded because of incomplete clinical or procedural data, which may have introduced selection bias and limits the generalisability of these findings. Additionally, the composite endpoint encompassed several types of clinical events, introducing heterogeneity. Detailed in-hospital procedural complications—including major coronary perforation, periprocedural myocardial infarction, emergency surgical revascularization, and hemodynamic compromise — were not systematically captured in our registry and could not be reliably adjudicated retrospectively across the 14-year study period. We therefore cannot determine the extent to which acute procedural complications contributed to the difference in long-term outcomes observed between successful and unsuccessful CTO PCI groups. This is an important question that warrants prospective evaluation in a contemporary cohort. Nonetheless, the use of bootstrap-derived confidence intervals strengthened the robustness of effect estimates in the presence of sparse data. Overall, the findings underscore the prognostic significance of CTO-related MI, procedural success, CKD, and LVEF in shaping long-term outcomes after CTO PCI (16).

## Conclusions

In Kaplan–Meier analysis, CTO-related myocardial infarction was associated with a higher cumulative incidence of adverse outcomes and unsuccessful CTO PCI with lower event-free survival; however, neither association reached statistical significance in bootstrap-adjusted multivariable analysis. Chronic kidney disease emerged as the strongest independent determinant of adverse outcomes, whereas higher left ventricular ejection fraction was protective (4). These findings suggest that patient substrate plays a central role in long-term risk, beyond CTO-specific anatomical considerations (17). Identifying high-risk subgroups and optimizing procedural success may help improve long-term outcomes in this complex patient population (3).

## Data Availability

The raw data supporting the conclusions of this article will be made available by the authors, without undue reservation.

## Supplementary material

Supplementary Figure S1 Multivariable Cox regression analysis for the composite endpoint. Forest plot of the multivariable Cox proportional hazards model for the composite endpoint. Hazard ratios with 95% confidence intervals were derived using bootstrap resampling (1,000 iterations). Chronic kidney disease showed the strongest independent association with adverse outcomes, whereas a higher left ventricular ejection fraction was protective.

Supplementary Figure S2 Kaplan–Meier curves for all-cause mortality and cardiovascular mortality stratified by CTO-related myocardial infarction (MI) status and procedural success. (A) All-cause mortality according to CTO-related MI status (log-rank *p* = 0.146). (B) All-cause mortality according to procedural success (log-rank *p* = 0.025). (C) Cardiovascular mortality according to CTO-related MI status (log-rank *p* = 0.410). (D) Cardiovascular mortality according to procedural success (log-rank *p* = 0.015). Event-free survival curves are shown for the overall cohort (*n* = 337 after exclusion of patients with non-positive follow-up time). Numbers at risk are displayed below each panel. These prespecified secondary analyses were performed to clarify the contribution of hard clinical endpoints to the primary composite outcome. Associations observed in unadjusted Kaplan–Meier analyses should be interpreted in the context of the multivariable-adjusted results presented in the main text.

Supplementary Figure S3 Kaplan–Meier and interaction analyses examining the association between procedural success and the composite endpoint according to chronic kidney disease (CKD) status. (A) Kaplan–Meier analysis of the composite endpoint among patients without CKD (*n* = 309), comparing successful and unsuccessful CTO PCI (log-rank *p* = 0.191). (B) Kaplan–Meier analysis among patients with CKD (*n* = 28), comparing successful and unsuccessful CTO PCI (log-rank *p* = 0.083). The small number of CKD patients (17 successful, 11 unsuccessful) limits statistical precision. (C) Multivariable Cox proportional hazards model including a prespecified CKD  ×  procedural success interaction term. The interaction term did not reach statistical significance (HR 2.94, 95% CI 0.72–11.99; *p* = 0.265), indicating no statistically significant evidence of effect modification by CKD status. Hazard ratios are shown on a logarithmic scale. These analyses were performed in response to concerns regarding potential confounding by baseline renal dysfunction and to explore whether the association between procedural success and long-term outcomes differed according to CKD status.
