# Supplementary material for: Impact of CTO-related myocardial infarction and procedural success on long-term outcomes after CTO PCI
Source: Front Cardiovasc Med. 2026 Jul 28;13:1846597. doi: 10.3389/fcvm.2026.1846597 (PMC13457022; doi:10.3389/fcvm.2026.1846597)
Supplement: Supplementary file 1 [file Table1.docx]

**Supplementary Table 1.** **Compared baseline clinical characteristics between the successful and unsuccessful CTO PCI groups**

| **Variable** | **Failed CTO PCI (n=100)** | **Successful CTO PCI (n=237)** | **p-value** |
| --- | --- | --- | --- |
| **Age, years** | 65.5 [57.0–74.0] | 62.0 [54.0–72.0] | 0.146 |
| **Male, n (%)** | 70 (70.0) | 163 (68.8) | 0.926 |
| **LVEF, %** | 52.2 [42.3–60.9] | 57.3 [45.6–63.1] | **0.021** |
| **Hemoglobin, g/dL** | 13.9 [12.1–14.9] | 13.6 [12.3–14.9] | 0.978 |
| **Creatinine, mg/dL** | 1.0 [0.8–1.2] | 0.9 [0.8–1.1] | 0.063 |
| **BUN, mg/dL** | 16.6 [13.8–21.4] | 16.6 [13.5–21.7] | 0.930 |
| **LDL-C, mg/dL** | 109 [79–136] | 104 [82–134] | 0.947 |
| **CRP, mg/dL** | 0.3 [0.1–1.1] | 0.3 [0.1–0.9] | 0.835 |
| **Hypertension, n (%)** | 61 (61.0) | 150 (63.3) | 0.784 |
| **Diabetes mellitus, n (%)** | 41 (41.0) | 96 (40.5) | 1.000 |
| **Current smoker, n (%)** | 46 (46.0) | 100 (42.2) | 0.600 |
| **Chronic kidney disease, n (%)** | 11 (11.0) | 17 (7.2) | 0.344 |
| **Peripheral artery disease, n (%)** | 1 (1.0) | 1 (0.4) | 0.506 |
| **COPD, n (%)** | 2 (2.0) | 2 (0.8) | 0.585 |
| **Prior CVA, n (%)** | 8 (8.0) | 23 (9.7) | 0.773 |
| **CTO-related MI, n (%)** | 33 (33.0) | 67 (28.3) | 0.461 |

*Continuous variables expressed as median [IQR]; categorical variables as n (%). Between-group comparisons used the Mann–Whitney U test and χ² or Fisher’s exact test as appropriate.*
